# Clinical assessment of endothelial function in convalescent COVID-19 patients: a meta-analysis with meta-regressions

**DOI:** 10.1080/07853890.2022.2136403

**Published:** 2022-11-16

**Authors:** Pasquale Ambrosino, Stefano Sanduzzi Zamparelli, Marco Mosella, Roberto Formisano, Antonio Molino, Giorgio Alfredo Spedicato, Antimo Papa, Andrea Motta, Matteo Nicola Dario Di Minno, Mauro Maniscalco

**Affiliations:** a Cardiac Rehabilitation Unit of Telese Terme Institute, Istituti Clinici Scientifici Maugeri IRCCS, Pavia, Italy; b Department of Clinical Medicine and Surgery, Federico II University, Naples, Italy; c Neurological Rehabilitation Unit of Telese Terme Institute, Istituti Clinici Scientifici Maugeri IRCCS, Pavia, Italy; d Department of Data Analytics and Actuarial Science, Unipol Group, Bologna, Italy; e Institute of Biomolecular Chemistry, National Research Council, Pozzuoli, Italy; f Pulmonary Rehabilitation Unit of Telese Terme Institute, Istituti Clinici Scientifici Maugeri IRCCS, Pavia, Italy

**Keywords:** Post-acute COVID-19 syndrome, long COVID, endothelial dysfunction, disability, exercise, rehabilitation

## Abstract

**Background:**

Endothelial dysfunction has been proposed to play a key role in the pathogenesis of coronavirus disease 2019 (COVID-19) and its post-acute sequelae. Flow-mediated dilation (FMD) is recognized as an accurate clinical method to assess endothelial function. Thus, we performed a meta-analysis of the studies evaluating FMD in convalescent COVID-19 patients and controls with no history of COVID-19.

**Methods:**

A systematic literature search was conducted in the main scientific databases according to the Preferred Reporting Items for Systematic Reviews and Meta-Analyses (PRISMA) guidelines. Using the random effects method, differences between cases and controls were expressed as mean difference (MD) with 95% confidence intervals (95% CI). The protocol was registered on PROSPERO with reference number CRD42021289684.

**Results:**

Twelve studies were included in the final analysis. A total of 644 convalescent COVID-19 patients showed significantly lower FMD values as compared to 662 controls (MD: −2.31%; 95% CI: −3.19, −1.44; *p* < 0.0001). Similar results were obtained in the sensitivity analysis of the studies that involved participants in either group with no cardiovascular risk factors or history of coronary artery disease (MD: −1.73%; 95% CI: −3.04, −0.41; *p* = 0.010). Interestingly, when considering studies separately based on enrolment within or after 3 months of symptom onset, results were further confirmed in both short- (MD: −2.20%; 95% CI: −3.35, −1.05; *p* < 0.0001) and long-term follow-up (MD: −2.53%; 95% CI: −4.19, −0.86; *p* = 0.003). Meta-regression models showed that an increasing prevalence of post-acute sequelae of COVID-19 was linked to a higher difference in FMD between cases and controls (Z-score: −2.09; *p* = 0.037).

**Conclusions:**

Impaired endothelial function can be documented in convalescent COVID-19 patients, especially when residual clinical manifestations persist. Targeting endothelial dysfunction through pharmacological and rehabilitation strategies may represent an attractive therapeutic option.

**Key messages**
- The mechanisms underlying the post-acute sequelae of coronavirus disease 2019 (COVID-19) have not been fully elucidated.
- Impaired endothelial function can be documented in convalescent COVID-19 patients for up to 1 year after infection, especially when residual clinical manifestations persist.
- Targeting endothelial dysfunction may represent an attractive therapeutic option in the post-acute phase of COVID-19.

## Introduction

The new severe acute respiratory syndrome coronavirus 2 (SARS-CoV-2) first appeared in eastern China in late 2019, leading to a global emergency and subsequent pandemic declaration in 2020 [1]. Although SARS-CoV-2 infection can occur asymptomatically, especially among vaccinated people [2], it can also determine coronavirus disease 2019 (COVID-19), which can give mild symptoms or, conversely, progress to acute respiratory distress syndrome and multiorgan failure [3].

From the early stages of the pandemic, the persistence of clinical and functional limitations after the acute phase of the disease quickly became evident, potentially involving multiple organs and domains [4]. Thus, a plethora of post-acute sequelae of COVID-19 (PASC) have been reported weeks to months after infection, including fatigue, shortness of breath, joint pain as well as psychological and even cognitive disorders [5]. Moreover, severe life-threatening complications have been documented up to 12 months after swab test negativization, with an increased risk of both venous and arterial thrombosis even among patients who had experienced only mild or moderate symptoms [6]. Thus, the need for multidisciplinary post-acute care and personalized rehabilitation programmes has progressively emerged [7,8].

To date, the pathophysiological mechanisms underlying such persistent or delayed clinical manifestations of the convalescent phase are still a matter of study [9]. If dysautonomia, immunological dysregulation or even persistent infection have often been considered [10], mounting evidence suggests that endothelial damage and subsequent endothelial dysfunction may represent the common background of most complications of the post-acute phase, including arterial and venous thrombosis [11,12]. Therefore, the European Society of Cardiology (ESC) has suggested the monitoring of endothelial function in the follow-up of convalescent COVID-19 patients for an early identification of such thrombotic complications [13].

Different laboratory and clinical methods are currently available to monitor endothelial function, with some relevant limitations in terms of invasiveness or costs [11]. To date, assessment of brachial artery flow-mediated dilation (FMD) and nitrate-mediated dilation (NMD) are considered reliable and non-invasive clinical methods for endothelial function assessment, particularly if performed with the aid of a Food and Drug Administration (FDA)-cleared software for shear-rate monitoring and wall-tracking [11]. Given the key role of endothelial integrity in vascular health, FMD has also become a surrogate marker of cardiovascular risk and an independent predictor of cardiovascular events [14].

Therefore, in line with ESC recommendation [13], some studies have evaluated FMD or NMD in COVID-19 survivors since the early stages of the pandemic, reporting reduced values compared to non-COVID-19 controls [15,16]. However, this result has been questioned in other articles [17,18], and no meta-analytical data summarizing the literature evidence on this topic have been provided so far.

In light of the above, we planned to perform a systematic review with meta-analysis of all studies evaluating FMD or NMD in convalescent COVID-19 patients and controls with no history of COVID-19. In addition, some meta-regression analyses were implemented to evaluate the impact of the main clinical and demographic variables on the observed results.

## Methods

For the systematic search of the literature, we designed a protocol with specific objectives and inclusion criteria, predetermining the outcomes, statistical analyses and methods of assessing the quality of the studies. The protocol was previously registered on PROSPERO with reference number CRD42021289684.

### Search strategy

We performed a systematic search of the literature in the main scientific databases (PubMed, Web of Science, Scopus and EMBASE), following the Preferred Reporting Items for Systematic Reviews and Meta-Analyses (PRISMA) guidelines [19]. Our search terms were (*COVID-19* or *long COVID* or *post-COVID-19* or *COVID long-haulers* or *severe acute respiratory syndrome coronavirus 2* or *SARS-CoV-2*) and (*flow-mediated dilation* or *FMD* or *nitrate-mediated dilation* or *NMD* or *endothelium-dependent dilation* or *endothelium-independent dilation*). To avoid unintentionally removing of articles, no filters or language restrictions were applied. We performed the last search on 9 June 2022 (Supplemental Table 1).

In addition, we reviewed the reference lists of the selected articles and, in case of studies that could potentially be included in the meta-analysis, we wrote to the authors to retrieve the missing data. The texts of the articles were evaluated by two independent investigators (MM and RF), who extracted the data separately. In case of disagreement between the two, a third investigator was also involved (AP). Overall, a high inter-reader agreement was achieved (*κ* = 0.97). The results of the literature search were detailed according to the PRISMA flowchart.

**Figure 1.**
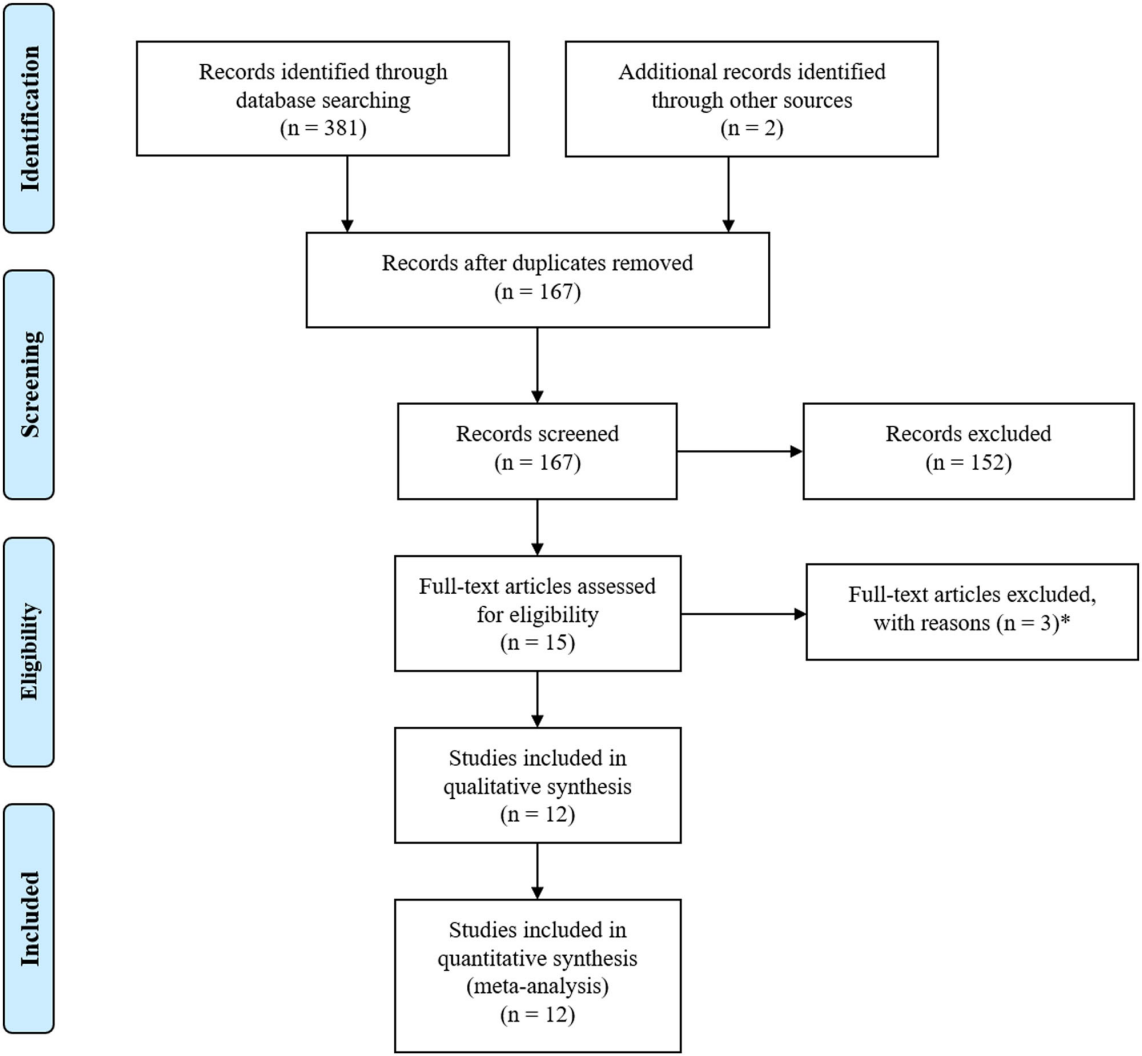
Preferred Reporting Items for Systematic Reviews and Meta-Analyses (PRISMA) flow diagram. *Two studies without control group, one study on acute coronavirus disease 2019 (COVID-19).

### Extraction of data and quality assessment

Following a pre-specified protocol, all studies evaluating FMD and/or NMD in convalescent COVID-19 and non-COVID-19 controls were considered. Case-series without a control group, case-reports, animal studies, posters from scientific conferences, and reviews were excluded. Overall, to enter the final analysis, a study had to report FMD and/or NMD values (expressed as means with standard deviations or standard errors) in COVID-19 survivors and in controls with no history of COVID-19. Wherever appropriate and applicable, data on sample size, FMD and NMD values, and major demographic or clinical variables related to comorbidities, therapies and the acute or convalescent phases of the infection were extracted in all included studies. Data on ultrasound equipment and medical software for the evaluation of endothelial function in each article were also collected.

The Newcastle–Ottawa Scale (NOS) for non-randomized observational studies was used to determine the methodological quality of the articles [20]. In brief, the scoring system evaluated 3 major areas: selection (4 records), comparability (1 record) and exposure (3 records). Each record received a maximum score of 1, except for comparability, as a maximum of 2 points could be awarded in this category. The total scores were calculated by adding the results of each record, with a final score ranging from a minimum of 0 (the lowest possible quality) to a maximum of 9 (the highest possible quality).

### Statistical analyses and assessment of bias

Statistical analyses were carried out with Comprehensive Meta-analysis version 3 (Biostat Inc., Englewood, NJ) and R Statistical software version 4.2.1 (R Foundation for Statistical Computing, Vienna, Austria). To document differences between cases and controls, mean differences (MDs) with 95% confidence intervals (95% CIs) were used. Prediction intervals, reflecting the range within which the results of a future study might lie, were also calculated [21]. To be as conservative as possible, the random effects method was used in all analyses, thus considering both within-study and between-study variance [22]. In case of non-independence of the effect sizes among the retrieved datasets, a multilevel approach was also adopted to refine and possibly confirm the results [23].

The overall effect was tested using Z-scores and a *p* ≤ 0.05 was presumed to be statistically significant. Statistical heterogeneity among studies was taken into account and calculated by using the chi-square Cochran’s Q test and the *I*^2^ index. The *I^2^* index measured the inconsistency among the study results, representing the proportion of total variation in study estimates due to heterogeneity rather than sampling error. Briefly, *I*^2^ values of 0% indicated the absence of heterogeneity while values below 25% suggested low heterogeneity, which became moderate from 25 to 50% and high for values greater than 50% [24]. In order to identify potential sources of any heterogeneity, we repeated the analyses after excluding one study at a time if a significant heterogeneity was found.

Funnel plots of the effect size *vs.* precision (1/standard error of the MD) were visually inspected to identify asymmetry and address for potential small-study effect. Additionally, the Egger’s regression test and the Begg and Mazumdar rank correlation test were used to assess publication bias, beyond any subjective assessment. A *p* < 0.10 was assumed to be statistically significant [25]. Finally, we used the Duval and Tweedie’s trim-and-fill analysis to calculate an adjusted effect size after trimming and imputing studies [26].

### Sensitivity analyses

In order to investigate potential sources of heterogeneity, we planned to repeat the analyses by including only ‘high quality’ studies according to NOS (i.e. NOS ≥ median value found among included studies). In addition, as FMD and NMD are widely accepted as surrogate markers of cardiovascular risk [14,27], another sensitivity analysis was planned for studies specifically excluding participants with traditional cardiovascular risk factors, such as diabetes or hypertension, or any history of coronary artery disease (CAD). Moreover, we planned to perform an analysis after eliminating the studies that enrolled paediatric populations. Given the potential operator dependence of FMD and NMD evaluation, we also decided to separately consider only the studies that used a dedicated software for wall tracking and shear-rate monitoring. Finally, considering the observational nature of the data, a sensitivity analysis for unmeasured confounding was also planned. Thus, the E*-*values for the point estimate and the lower confidence interval limit were calculated to estimate the robustness of the results against unmeasured confounders on the risk ratio scale [28].

### Subgroup analyses

Considering the high heterogeneity between studies in the duration of the follow-up period, we planned to separately analyse those evaluating convalescent patients within the first 3 months of SARS-CoV-2 infection or, conversely, long after recovery (> 3 months).

### Meta-regression analyses

We also speculated that our results on FMD may be influenced by the differences between cases and controls in major demographic (male gender, mean age) and clinical variables related to cardiovascular risk (hypertension, hypercholesterolaemia, diabetes, smoking, body mass index [BMI] and history of CAD) and use of cardiovascular medications (calcium channel blockers [CCB], β-blockers, diuretics, rennin angiotensin system [RAS] inhibitors, statins, antiplatelets, anticoagulants, oral antidiabetics, and insulin). Similarly, we considered the possibility that the difference in clinical measures of endothelial function between cases and controls could be influenced by a number of variables related to the severity of COVID-19 during the acute phase (critical illness, hospitalization, length of hospital stays, need for high-flow oxygen (O_2_) or mechanical ventilation, access to the intensive care unit [ICU]). Finally, we tested the hypothesis that our results may be affected by any residual laboratory alterations or clinical manifestations of convalescence (white blood cell [WBC] count, C-reactive protein [CRP] levels and prevalence of PASC).

In order to test the possible effect of the above variables in explaining the results observed across studies, we planned to perform meta-regression analyses after implementing regression models with differences in FMD or NMD as dependent variables (y) and these covariates as independent ones (x). For multivariable meta-regressions, multicollinearity was quantified by means of the variance inflation factor (VIF), with values greater than 2.5 indicating levels of collinearity that could negatively impact the regression model [29].

## Results

After eliminating duplicate results, 167 articles were considered. Of these, we excluded 94 as irrelevant after scanning the title and/or the abstract, and 58 comments/case reports/reviews or studies with no data of interest. Another three studies were discarded after full-text evaluation.

As a result, we considered 12 articles for the final analysis [15–18,30–37] (Figure 1). All the retrieved studies contained data on FMD, spread across 17 datasets on a total of 644 convalescent COVID-19 patients and 662 controls. On the other hand, only one article [17] also reported on NMD, so no meta-analytical evaluation could be performed for the latter outcome.

### Study characteristics

The studies included in the meta-analysis had a case-control design. The major demographic and clinical characteristics of the study populations have been reported in Table 1. In the retrieved datasets, the number of convalescent COVID-19 patients ranged from 8 to 133, with a mean age varying between 8.9 and 68.7 years, mean BMI between 19.6 and 29.4 kg/m,^2^ and male gender prevalence between 12.5 and 81.2%. Diabetes was documented in 0–25.9% of patients, hypertension in 0–51.1%, and dyslipidaemia in 0–42.9%. The prevalence of CAD ranged between 0 and 15.1%, while a smoking history was reported by 0–42.9% of patients.

**Table 1.** Demographic and clinical data of convalescent coronavirus disease 2019 (COVID-19) patients and controls in included datasets.

Study	Subjects (*n*)	Males *n* (%)	Age (years)	BMI (kg/m^2^)	Diabetes *n* (%)	Hypertension *n* (%)	Hypercholesterolaemia *n* (%)	Smoking^a^ *n* (%)	SBP (mmHg)	DBP (mmHg)	CAD *n* (%)
Ambrosino et al. [4]	133 COVID-19	108 (81.2)	61.6	–	21 (15.8)	68 (51.1)	12 (9.0)	12 (9.0)	–	–	19 (14.3)
	133 Controls	107 (80.5)	60.4	–	23 (17.3)	74 (55.6)	14 (10.5)	12 (9.0)	–	–	24 (18.0)
Çiftel et al. [31]	38 COVID-19	20 (52.6)	8.9	19.6	0	0	0	0	99.6	61.5	0
	38 Controls	20 (52.6)	8.9	20.1	0	0	0	0	108.9	68.2	0
Ergül et al. [32]	63 COVID-19	–	–	–	–	–	–	–	–	–	–
	29 Controls	–	–	–	0	0	0	–	–	–	0
Gao et al. [33] (a)	86 COVID-19	32 (37.2)	55.7	24.0	14 (16.3)	32 (37.2)	16 (18.6)	–	131.0	76.3	13 (15.1)
	28 Controls	10 (35.7)	52.7	23.0	0	0	0	–	125.0	74.0	0
Gao et al. [33] (b)	86 COVID-19	32 (37.2)	55.7	24.0	14 (16.3)	32 (37.2)	16 (18.6)	–	131.0	76.3	13 (15.1)
	30 –Controls	11 (36.7)	58.7	24.0	2 (6.7)	10 (33.3)	9 (30.0)	–	126.0	72.7	3 (10.0)
Jud et al. [17] (a)	14 COVID-19	7 (50.0)	68.7	29.4	0	6 (42.9)	6 (42.9)	6 (42.9)	–	–	0
	14 controls	7 (50.0)	30.7	23.8	0	0	0	8 (57.1)	–	–	0
Jud et al. [17] (b)	14 COVID-19	7 (50.0)	68.7	29.4	0	6 (42.9)	6 (42.9)	6 (42.9)	–	–	0
	14 Controls	7 (50.0)	66.9	27.6	4 (28.6)	13 (92.9)	12 (85.7)	11 (78.6)	–	–	8 (57.1)
Lambadiari et al. [34] (a)	70 COVID-19	44 (62.9)	54.5	–	0	0	0	16 (22.9)^c^	129.7	78.2	0
	70 Controls	44 (62.9)	54.8	–	0	0	0	21 (30.0)^c^	126.6	80.7	0
Lambadiari et al. [34] (b)	70 COVID-19	44 (62.9)	54.5	–	0	0	0	16 (22.9)^c^	129.7	78.2	0
	70 Controls	44 (62.9)	54.5	–	0	70 (100)	0	18 (25.7)^c^	145.3	89.9	0
Mansiroglu et al. [37]	80 COVID-19	32 (40.0)	32.1	25.6	2 (2.5)	2 (2.5)	1 (1.3)	19 (23.8)	105.0	70.0	0
	81 Controls	41 (44.4)	30.5	20.0	2 (2.5)	2 (2.5)	1 (1.2)	23 (28.4)	110.0	70.0	0
Nandadeva et al. [35] (a)	8 COVID-19	1 (12.5)	24.0	26.0	0	0	0	0	111.0	70.0	0
	12 Controls	6 (50.0)	23.0	23.0	0	0	0	0	112.0	66.0	0
Nandadeva et al. [35] (b)	8 COVID-19	5 (62.5)	22.0	22.0	0	0	0	0	110.0	68.0	0
	12 controls	6 (50.0)	23.0	23.0	0	0	0	0	112.0	66.0	0
Oikonomou et al. [15] (a)	55 COVID-19	32 (58.2)	57.8	–	12 (21.8)	21 (38.2)	14 (25.5)^b^	–	129.0	80.0	3 (5.5)
	55 Controls	29 (52.7)	62.6	–	17 (30.9)	28 (50.9)	15 (27.3)^b^	–	135.0	80.0	3 (5.5)
Oikonomou et al. [15] (b)	55 COVID-19	32 (58.2)	57.8	–	12 (21.8)	21 (38.2)	14 (25.5)^b^	–	132.0	81.0	3 (5.5)
	55 Controls	29 (52.7)	62.6	–	17 (30.9)	28 (50.9)	15 (27.3)^b^	–	135.0	80.0	3 (5.5)
Ratchford et al. [16]	11 COVID-19	4 (36.4)	20.1	23.5	0	0	0	0	121.3	71.8	0
	20 Controls	5 (25.0)	23.0	22.5	0	0	0	0	111.8	77.7	0
Riou et al. [36]	27 COVID-19	17 (63.0)	57.3	29.7	7 (25.9)	13 (48.1)	–	6 (22.2)	134.0	84.7	–
	9 Controls	5 (55.6)	58.3	–	–	–	–	–	–	–	–
Skow et al. [18]	23 COVID-19	9 (39.1)	23.0	25.3	0	0	0	0	109.0	67.0	0
	13 Controls	6 (46.1)	26.0	25.7	0	0	0	0	113.0	69.0	0

BMI: body mass index; SBP: systolic blood pressure; DBP: diastolic blood pressure; CAD: coronary artery disease.

Continuous data are reported as mean values, unless otherwise indicated. The minus sign indicates that the information has not been specifically provided and/or cannot be inferred from the text of the article.

^a^Any smoking history.

^b^Dyslipidaemia.

^c^Current smokers.

The severity of COVID-19 during the acute phase varied widely between studies, being hospitalization reported in 0–100% of patients with a mean length of hospital stays ranging from less than 15 to 25.4 days. Moreover, 0–69.2% of patients had experienced a critical disease, with 0–40.7% requiring intensive care. Thus, high-flow O_2_ was needed in up to 26.3% of patients, while the use of mechanical ventilation was reported in up to 27.1% of cases. When considered after the acute phase, the frequency of any residual clinical manifestation related to COVID-19 was reported in 0–100% of cases, with mean WBC counts ranging from 6.1 to 14.0 × 10^9^/L and mean CRP levels from 1.3 to 152.0 mg/L (Table 2). Data on the use of cardiovascular medications at the time of testing were omitted in most studies, with the use of β-blockers being reported in up to 31.6% of COVID-19 patients, CCB in up to 14.3%, RAS inhibitors in up to 35.7%, diuretics in up to 7.1%, statins in up to 29.3%, antiplatelets in up to 15.0%, anticoagulants in up to 14.3%, insulin in up to 12.8%, and oral antidiabetics in up to 12.0% (Supplemental Table 2). The time after the acute phase during which patients were included in the studies ranged from a few weeks to 1 year. With the only exception of one study [18] carried out in 2022, all included studies enrolled patients until June 2021 at the latest (Supplemental Table 3).

**Table 2.** Disease severity and post-acute sequelae of coronavirus disease 2019 (COVID-19) in included datasets.

		Acute phase						Convalescent phase		
Study	Patients (*n*)	Critical disease^a^ *n* (%)	Hospitalization *n* (%)	LHS (days)	High-flow O_2_ *n* (%)	Mechanical ventilation *n* (%)	ICU *n* (%)	WBC (10^9^/L)	CRP (mg/L)	PASC^b^ *n* (%)
Ambrosino et al. [4]	133	92 (69.2)	94 (70.7)	25.4	35 (26.3)	36 (27.1)	92 (69.2)	–	–	133 (100)
Çiftel et al. [31]	38	–	–	–	–	–	–	14.0	152.0	38 (100)
Ergül et al. [32]	63	–	63 (100)	–	–	–	–	–	–	–
Gao et al. [33] (a)	86	14 (16.3)	78 (90.7)	–	–	6 (7.0)	–	–	1.3	–
Gao et al. [33] (b)	86	14 (16.3)	78 (90.7)	–	–	6 (7.0)	–	–	1.3	–
Jud et al. [17] (a)	14	3 (21.4)	14 (100)	–	–	–	–	6.1	37.0	–
Jud et al. [17] (b)	14	3 (21.4)	14 (100)	–	–	–	–	6.1	37.0	–
Lambadiari et al. [34] (a)	70	0	46 (65.7)	< 15	–	0	0	–	–	26 (37.9)
Lambadiari et al. [34] (b)	70	0	46 (65.7)	< 15	–	0	0	–	–	26 (37.9)
Mansiroglu et al. [37]	80	0	0	–	0	0	0	−	–	0
Nandadeva et al. [35] (a)	8	–	−	−	−	−	−	−	–	8 (100)
Nandadeva et al. [35] (b)	8	–	−	−	−	−	−	−	–	0
Oikonomou et al. [15] (a)	55	–	−	−	−	−	−	−	4.0	–
Oikonomou et al. [15] (b)	55	–	−	−	−	−	−	−	2.0	32 (58.2)
Ratchford et al. [16]	11	–	−	−	−	−	−	−	−	10 (90.9)
Riou et al. [36]	27	11 (40.7)^c^	27 (100)	21.3	−	−	11 (40.7)	−	−	−
Skow et al. [18]	23	0	0	–	0	0	0	−	−	4 (17.4)

LHS: length of hospital stays; ICU: intensive care unit; WBC: white blood cells; CRP: C-reactive protein; PASC: post-acute sequelae of COVID-19

The minus sign indicates that the information has not been specifically provided and/or cannot be inferred from the text of the article.

^a^Critical disease according to World Health Organization (WHO) and/or National Institute of Health (NIH) disease severity classification for COVID-19 (i.e. acute respiratory distress syndrome, sepsis, septic shock, acute arterial or venous thromboembolism and multiple organ dysfunction).

^b^At study entry, any persistent clinical manifestation after SARS-CoV-2 infection, including delayed syndromes (e.g. multisystem inflammatory syndrome in adults and children).

^c^Severe to critical patients according to WHO disease severity classification.

Among the considered studies, three [17,33,34] decided to evaluate endothelial function in convalescent COVID-19 patients and in two separate control groups, one of which consisted of healthy non-COVID-19 participants. Thus, Gao et al. [33] also included a control group matched for the major cardiovascular risk factors, while another study [17] considered further controls with atherosclerotic cardiovascular disease (at least one among CAD, cerebrovascular disease, lower extremity arterial disease, and upper extremity arterial disease). Similarly, Lambadiari et al. [34] examined an additional control group of hypertensive otherwise healthy subjects. On the other hand, cases and controls were tested at two different time-points in another article [15], namely 1 and 6 months after hospital discharge. Finally, Nandadeva et al. [35] separately reported data on FMD in COVID-19 patients based on the presence/absence of any residual clinical manifestation after the acute phase, with a partial overlap of the control population with that included in another study [18]. In all these cases, different datasets were considered for the analyses and no selection scheme was adopted, while also taking a multilevel approach to manage the nested structure of the datasets and draw more robust conclusions.

Although only four studies [16,18,30,35] used a dedicated edge detection software, all used the same clinical method for endothelial function assessment and all followed the same standardized procedures [38] (Supplemental Table 4).

The NOS for quality assessment of included studies had a median value of 7 (Supplemental Table 5).

### Meta-analysis of flow-mediated dilation (FMD) and publication bias

**Figure 2.**
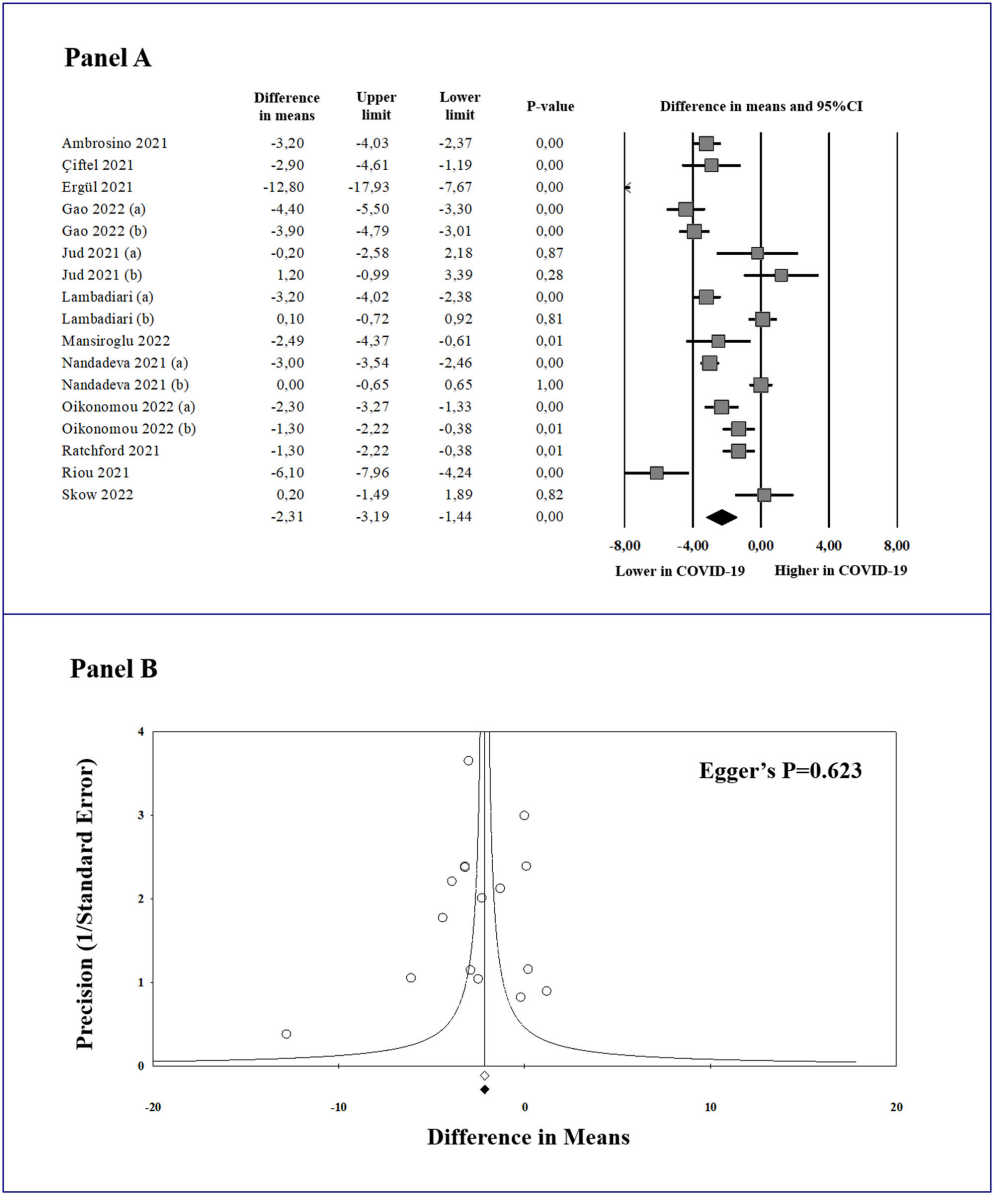
Forest plot of the mean difference in flow-mediated dilation (FMD) between convalescent coronavirus disease 2019 (COVID-19) patients and controls (A), and funnel plot of effect size *vs.* precision (1/standard error of the mean difference) for studies evaluating FMD in cases and controls (B). 95% CI: 95% confidence interval. In Panel B, observed studies and effect size are represented by empty circles and empty square. Imputed studies and adjusted effect size are represented by black circles and black square.

In 12 studies (17 datasets) [15–18,30–37], a total of 644 convalescent COVID-19 patients showed significantly lower FMD values as compared to 662 controls (MD: −2.31%; 95% CI: −3.19, −1.44; *p* < 0.0001, Figure 2(A)). Computing the prediction interval, we calculated that the true effect size in 95% of future comparable populations would fall between −6.00 and 1.37. A significant heterogeneity among the studies was found (*I*^2^ = 91.3%; *p* < 0.0001), which remained substantially unchanged after excluding one study at a time. Interestingly, considering the non-independence of the effect sizes with a multilevel approach, the above results were largely confirmed (MD: −2.29%; 95% CI: −3.36, −1.22; *p* < 0.0001), with high total heterogeneity (*I*^2^ = 98.6%; *p* < 0.0001) and a similar prediction interval (between −6.10 and 1.52).

Given the potential influence of publication bias on meta-analyses results, we visually inspected the funnel plot of effect size *vs.* precision (1/standard error of the MD) for studies evaluating FMD in order to identify asymmetry (Figure 2(B)). Being rather symmetrical, we excluded the presence of any publication bias and small-study effect, confirmed by the Egger’s regression test (*p* = 0.623) and the Begg and Mazumdar rank correlation test (*p* = 0.592). Accordingly, the Duval and Tweedie’s trim and fill analysis showed that, after trimming and imputing studies, all results were substantially verified (Supplemental Table 6).

### Sensitivity analyses

Given the median value of the NOS quality assessment of 7 (Supplemental Table 5), we repeated the analyses by including only the studies with a score ≥ 7 [15,18,30–32,34,37]. Interestingly, when separately considering these ‘high quality’ studies, our results on FMD were substantially confirmed (MD: −2.30%; 95% CI: −3.45, −1.15; *p* < 0.0001, Table 3(A)). A significant difference in FMD was also obtained by analysing separately the studies [16,18,31,34,35] that involved participants in either group with no cardiovascular risk factors or history of CAD (Table 3(B)) and, furthermore, by excluding any article [31] on paediatric populations (Table 3(C)). Finally, when analysing only the studies [16,18,30,35] that used a semi-automatic software to increase reproducibility of FMD assessment, a significant difference between convalescent COVID-19 patients and controls was further confirmed (Table 3(D)). Interestingly, assuming the non-independence of the effect sizes, the multilevel analyses substantially confirmed the above results (Supplemental Table 7).

**Table 3.** Sensitivity and subgroup analyses for studies evaluating flow-mediated dilation (FMD) in convalescent coronavirus disease 2019 (COVID-19) patients and controls.

	*N* of studies	*N* of datasets	*N* of patients	Effect size
Sensitivity analyses	A. ‘High quality’ studies			
	7	9	517 COVID-19 489 controls	MD: −2.30 (95% CI: −3.45, −1.15); *p* < 0.0001 *I*^2^ = 88.4%; *p* < 0.0001 PI: −6.25, 1.66
	B. Exclusion of participants with cardiovascular risk factors or history of events			
	5	6	202 COVID-19 188 controls	MD: −1.73 (95% CI: −3.04, −0.41); *p* = 0.010 *I*^2^ = 92.5%; *p* < 0.0001 PI: −6.38, 2.93
	C. Exclusion of paediatric populations			
	11	16	606 COVID-19 624 controls	MD: −2.28 (95% CI: −3.19, −1.37); *p* < 0.0001 *I*^2^ = 91.8%; *p* < 0.0001 PI: −6.04, 1.48
	D. Use of an automatic edge detection software			
	4	5	227 COVID-19 213 controls	MD: −1.53 (95% CI: −2.98, −0.07); *p* = 0.040 *I*^2^ = 93.9%; *p* < 0.0001 PI: −7.09, 4.04
Subgroup analyses	E. Follow-up ≤ 3 months			
	9	11	408 COVID-19 383 controls	MD: −2.20 (95% CI: −3.35, −1.05); *p* < 0.0001 *I*^2^ = 91.2%; *p* < 0.0001 PI: −6.37, 1.97
	F. Follow-up > 3 months			
	3	5	211 COVID-19 253 controls	MD: −2.53 (95% CI: −4.19, −0.86); *p* = 0.003 *I*^2^ = 94.1%; *p* < 0.0001 PI: −8.98, 3.93

Panel A: ‘high quality’ studies (Newcastle–Ottawa Scale ≥ 7); Panel B: studies specifically excluding participants with any cardiovascular risk factor or history of coronary artery disease; Panel C: exclusion of studies on paediatric populations; Panel D: studies using an automatic edge detection software for FMD assessment; Panel E: studies evaluating convalescent patients within the first 3 months of severe acute respiratory syndrome coronavirus 2 (SARS-CoV-2) infection; Panel F: studies evaluating COVID-19 participants more than 3 months after recovery.

*N*: number; MD: mean difference; 95% CI: 95% confidence interval; PI: prediction interval.

On average, an unmeasured confounder should have a minimum strength of association with both the outcome and the exposure of 3.73 on the risk ratio scale to nullify the observed effect size (Supplemental Figure 1). The E-value for the lower confidence limit was 2.60.

### Subgroup analyses

Considering the high heterogeneity between studies in the duration of the follow-up period (Supplemental Table 3), we separately analysed those evaluating COVID-19 patients within the first 3 months of SARS-CoV-2 infection [15,16,18,30–32,35,36] or, conversely, more than 3 months after recovery [15,33,34]. Interestingly, also excluding one study [37] for which the enrolment period was too heterogenous (less than 1 month to more than 6 months after infection), a significant difference in FMD between cases and controls was documented in both short- (MD: −2.20%; 95% CI: −3.35, −1.05; *p* < 0.0001, Table 3(E)) and long-term follow-up (MD: −2.53%; 95% CI: −4.19, −0.86; *p* = 0.003, Table 3(F)), confirmed when adopting a multilevel approach (Supplemental Table 7).

### Meta-regressions

**Figure 3.**
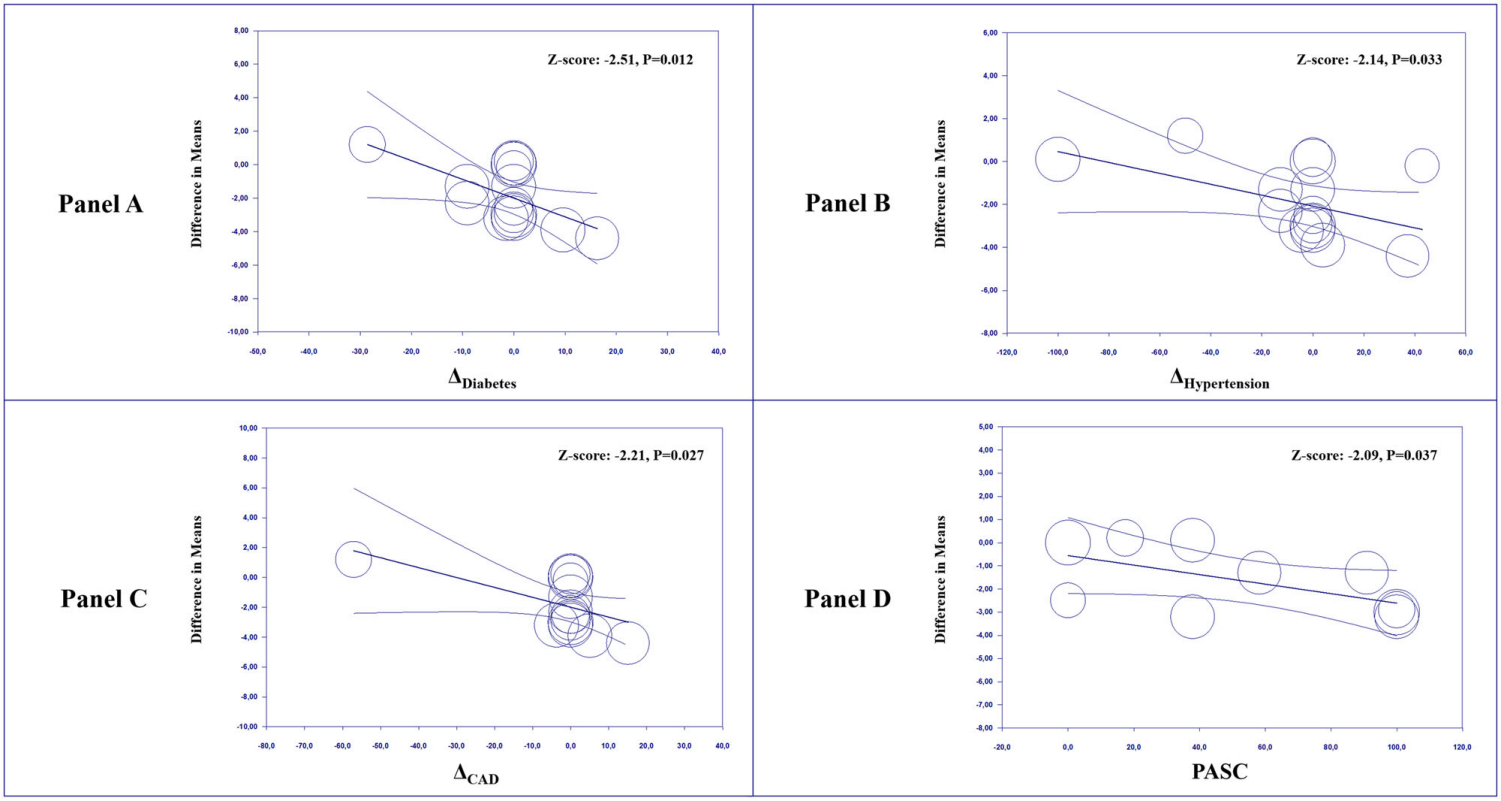
Meta-regression analyses. Impact of differences (Δ) in the prevalence of diabetes (Panel A), hypertension (Panel B), and coronary artery disease (Panel C) and impact of the post-acute sequelae of COVID-19 (Panel D) on the difference in flow-mediated dilation (FMD) between cases and controls. COVID-19: coronavirus disease 2019; PASC: post-acute sequelae of COVID-19.

Meta-regression analyses showed that, when comparing convalescent COVID-19 patients and controls, higher differences in the prevalence of diabetes (Z-score: −2.51; *p* = 0.012, Figure 3(A)), hypertension (Z-score: −2.14; *p* = 0.033, Figure 3(B)) and CAD (Z-score: −2.21; *p* = 0.027, Figure 3(C)) were associated with a larger effect size. In addition, an increasing prevalence of PASC was related to a higher difference in FMD between cases and controls (Z-score: −2.09; *p* = 0.037, Figure 3(D)). None of the other tested predictors impacted our findings in univariate meta-regressions (Supplemental Table 8).

In multivariable regression models, assuming that the differences between cases and controls in age, gender, and BMI were held constant, we found that the differences in the prevalence of diabetes (Z-score: −3.62; *p* < 0.001), hypertension (Z-score: −2.56; *p* = 0.011) and CAD (Z-score: −2.16; *p* = 0.031) were confirmed as independent predictors of the effect size (Supplemental Table 9), with multicollinearity being substantially excluded as a high VIF (i.e. ≥2.5) was not detected for any predictor in any of the tested models. No other multiple regression model could be implemented due to the lack of complete information on all predictors in included studies.

## Discussion

Supported by a number of sensitivity and subgroup analyses, results of this meta-analysis suggest that convalescent COVID-19 patients may have impaired endothelial function, as expressed by lower FMD values when compared to controls. A significant difference between cases and controls was found when analysing only the studies that specifically involved participants with no cardiovascular risk factors or history of CAD. Moreover, when considering studies separately based on enrolment within or after 3 months of infection, results were further confirmed in both short- and long-term follow-up. Finally, regression models showed that an increasing prevalence of PASC may be associated to a higher difference in FMD between cases and controls.

Taken together, these findings confirm and extend the growing body of scientific evidence on the potential role of endothelial dysfunction as a key pathogenic mechanism of COVID-19 and its post-acute sequelae [11,38,39]. It is now known that a number of residual clinical manifestations may persist beyond 4 weeks from symptom onset, even among patients experiencing only a mild or moderate disease [5], thus suggesting the need for multidisciplinary post-acute care and personalized rehabilitation programmes [40,41]. A recent meta-analysis concluded that the five most common symptoms up to 110 days after recovery are fatigue, headache, attention disorder, hair loss and dyspnoea [42]. Most importantly, residual computed tomography (CT) lesions could still be observed at 1-year follow-up, correlated with the impairment of functional parameters and lung volumes [43]. Fortunately, a continuous improvement of ventilated parenchyma and fibrotic-like CT alterations has been also documented after 12 [43] and 18 months [44]. However, the recent epidemiological data of an increased risk of arterial and venous thrombotic events up to 12 months after recovery, even among non-hospitalized patients [6], supported the urgent need to elucidate the putative mechanisms of such polymorphic delayed manifestations of COVID-19 [12].

In this regard, several mechanisms have been hypothesized to have a role in the pathogenesis of PASC and long-term thrombotic complications, including immune activation [45], persistent SARS-CoV-2 infection [46], reactivation of latent viruses [47], prolonged inflammation [48] and intense cardiopulmonary deconditioning [49]. However, from the earliest stages of the pandemic, it seemed clear that COVID-19 could ultimately be an endothelial disease [39]. Varga et al. were among the first to carry out the histopathological analysis of autopsy samples, demonstrating the presence of SARS-CoV-2 inside the endothelial cells of the lung with microvascular lymphocytic endotheliitis [50], later confirmed by other authors not only in the lung but also in the heart, kidneys, skin and even the reproductive system [51,52]. By directly infecting endothelial cells, it has been proposed a virus-induced down-regulation of angiotensin-converting enzyme 2 (ACE2), due to the endocytosis of the enzyme along with the viral particles and to the up-regulation of a disintegrin and metalloproteinase 17 (ADAM17), delegated to the proteolytic degradation of ACE2 [53,54]. In addition to being the gateway for SARS-CoV-2 to human cells, ACE2 is also the major angiotensin II-degrading enzyme, exerting its procoagulant, proinflammatory, and prooxidative effect *via* the angiotensin receptor type 1 (AT1) [30]. Beyond the direct viral cytopathic effect on endothelial cells, which has been recently questioned [55], it has been shown that inflammatory cytokines produced by activated leukocytes are capable of stimulating specific receptors on the surface of endothelial cells [11]. Among the inflammatory cytokines, interleukin-6 (IL6) mainly acts through Janus kinase/signal transducer and activator of transcription (JAK/STAT) activation [56], while the transcriptional activity of tumour necrosis factor-α (TNF-α) substantially depends on nuclear factor-κB (NF-κB) [57]. Although with different pathways, inflammatory cytokines act on endothelial cells by increasing the expression of several adhesion molecules and coagulation factors, including E-selectin, P-selectin, vascular cell adhesion molecule-1 (VCAM-1), intercellular adhesion molecule-1 (ICAM-1), von Willebrand factor (vWF) and tissue factor (TF) [58–60], while reducing nitric oxide (NO) bioavailability and increasing oxidative stress *via* activation of nicotinamide adenine dinucleotide phosphate (NADPH) oxidase [11]. All this results in reduced vasodilation, leukocyte adhesion and extravasation, platelet activation, amplification of primary haemostasis and activation of the extrinsic pathway of coagulation [61,62]. Whether the above mechanisms, that have been well studied in the acute phase of COVID-19, may persist weeks or months after infection has been a matter of debate [11]. Recently, it has been proposed that persistent or residual endotheliopathy may underlie most of the pleiotropic manifestations of long COVID [12,63], such as cognitive decline [64] and reduced exercise performance [65]. Accordingly, it has been demonstrated that endothelial dysfunction, hypercoagulability, and inflammation may be still detectable up to 1 year after recovery from COVID-19, as expressed by increased circulating levels of endothelin-1, ICAM-1, IL6, vWF, D-dimer and coagulation factor VIII [66,67].

### Potential clinical implications

Overall, our results are in line with this large amount of evidence supporting the potential role of endothelial dysfunction as a key pathogenic mechanism of COVID-19 and post-acute COVID-19 syndrome [11,38,39]. Our finding of a persistently impaired FMD in convalescent COVID-19 patients, confirmed in both short- and long-term follow-up, may be consistent with the evidence of an increased arterial and venous thrombotic risk after 1 year of acute infection [6]. This may be better understood if we consider the potential clinical relevance of FMD, which is the percentage dilation of the brachial artery on ultrasound after an ischaemic stimulus induced by the inflation of a pressure cuff placed on the forearm [68]. It has been reported that FMD in healthy individuals has an average value of 6.4%, with a significant age-dependent decline [69]. Considering that each percentage point decrease in FMD is associated with an increase of up to 13% in major adverse cardiovascular events [14,27], endothelium-dependent FMD has been widely accepted as a surrogate marker of cardiovascular risk. In our meta-analysis, the difference in FMD between cases and controls is very similar to that reported for other chronic conditions of different aetiology [70–72], thus suggesting that convalescent COVID-19 patients may exhibit a residual cardiovascular risk which may be somewhat comparable to that of chronic respiratory, endocrine and rheumatic diseases.

The evidence from our regression models of a higher effect size linked to a higher prevalence of PASC contrasts with the hypothesis of a dissociation between reported symptoms and objective measures of cardiopulmonary health during convalescence [73,74], suggesting instead that the ongoing mechanisms underlying symptoms may be somehow responsible for the reduced vascular reactivity. Therefore, beyond highlighting the limitations of routine clinical investigations in the post-acute phase of COVID-19 [63], our results may further support the potential role of endothelial dysfunction in determining or at least contributing to the clinical manifestations of convalescence.

In line with the evidence that diabetes and hypertension are strong cardiovascular risk factors [75], another finding of our regression models is that a higher difference in the prevalence of such variables or CAD may be associated to a higher difference in FMD between cases and controls. This may be due to a further increase in inflammation and oxidative stress induced by traditional cardiovascular risk factors [76], which also happen to be independent risk factors for a worse prognosis in SARS-CoV-2 infected patients [77,78]. Interestingly, although with a slightly smaller effect size, our results on FMD were substantially confirmed by analysing separately the studies that specifically involved participants with no cardiovascular risk factors or history of CAD in either group.

The clinical relevance of our findings should also be interpreted from a different point of view that, although in line with ESC recommendations [13], may go beyond the simple monitoring of endothelial function and cardiovascular risk in convalescent COVID-19 patients. In particular, if endothelial dysfunction has shown to play such a relevant role in this clinical setting, it may also be speculated that it is an attractive therapeutic target [11]. Two main classes of drugs have previously shown to positively impact endothelial function, namely statins and RAS inhibitors [79]. Although conflicting results have been reported so far [11], there is meta-analytical evidence that chronic statin use may be associated with a lower mortality in COVID-19 patients [80,81]. Similarly, although initially indicated as being responsible for greater susceptibility to the infection, RAS inhibitors also showed to reduce the risk of death in hospitalized COVID-19 patients [82]. Among the non-pharmacological approaches, rehabilitation has already demonstrated its usefulness in COVID-19 following the acute phase of the disease, being able to improve symptoms, quality of life, physical performance and lung functional parameters [83,84]. Since the first report in 1986 [85], several mechanisms have been hypothesized to explain the beneficial effects of rehabilitation and exercise-based interventions on endothelial function, including the reduction of inflammation and oxidative stress and the mobilization of endothelial progenitor cells [86]. Thus, in line with previous evidence in healthy subjects and cardiopulmonary diseases (e.g. chronic obstructive pulmonary disease and heart failure) [87–90], we were the first to demonstrate the potential beneficial effect of exercise-based rehabilitation on FMD also in convalescent COVID-19 patients [91]. While our meta-regressions do not seem to suggest any impact of any drug therapy on FMD of these patients, further studies with a robust design are needed to clarify whether specific pharmacological or rehabilitation strategies may be able to reduce endothelial dysfunction, thus potentially improving the cardiovascular risk profile of COVID-19 survivors.

### Limitations

Some potential limitations of our study should be addressed. First, since meta-analyses are performed on aggregate data, the use of regression models may help refine results, evaluating the influence of some clinical and demographic confounders on the effect size. However, heterogeneity among the studies was generally significant in our meta-analysis, thus caution should be taken in overall results interpretation. On the other hand, it is important to highlight that our findings were substantially confirmed in a number of appropriate sensitivity and subgroup analyses, with publication bias being consistently excluded by using different methods. Furthermore, the studies included in the meta-analysis contained data on the outcome of interest spread across 17 datasets, all of which were utilized so that our meta-analysis did not miss any opportunity to use the available data addressing the research question. Although this may lead to an overestimation of the pooled effect size, it is important to highlight that averaging or selecting effect sizes within studies are approaches which may have other relevant limitations [23]. Interestingly, our findings were always confirmed when adopting a multilevel approach that allowed to consider the nested structure of the effect sizes [23], thus giving a certain robustness to our results. A further methodological limitation that should be addressed refers to the observational nature of the available data. Although the prediction intervals always included the null effect in all analyses, it is noteworthy that the E-values for the point estimate and the confidence interval limit were not definitely small, thus suggesting a mild/moderate degree of robustness. On the other hand, a large amount of evidence in the literature [92] has shown that a number of variables could lead to a similar or even greater impairment of FMD with a risk ratio to be expected higher than 3.73. Thus, great caution is required when interpreting the results of our meta-analysis. Another relevant aspect is that FMD assessment may be influenced by many confounding factors, potentially limiting reproducibility of this technique. However, the full-text analysis of the included studies suggests that all used the same clinical method for endothelial function assessment and all followed the same standardized procedures [38]. Moreover, the sensitivity analysis of the studies that, to further increase reproducibility, used an automatic edge detection software substantially confirmed a significant difference in FMD between cases and controls. Another relevant limitation of our meta-analysis is that, although supported by pertinent sensitivity and subgroup analyses, our findings cannot be generalized to all SARS-CoV-2 variants and degrees of vaccination coverage. In fact, with the only exception of one study [18] carried out in 2022 during the Omicron wave in fully vaccinated participants, all included studies enrolled patients until June 2021 at the latest, with no information on SARS-CoV-2 variants or vaccination status. Thus, considering that most of the included studies were carried out between the end of 2020 and the first half of 2021, it is reasonable to assume that our results are limited to unvaccinated patients, most of which may have been infected with the Delta variant.

## Conclusions

In conclusion, impaired endothelial function can be documented in convalescent COVID-19 patients up to 1 year after infection, especially when residual clinical manifestations persist. Although the observational nature of the analysed data suggests great caution, these results may at least in part confirm and extend the recommendation for rigorous monitoring of cardiovascular risk and endothelial function in the post-acute phase of the disease. Targeting endothelial dysfunction through pharmacological strategies and multidisciplinary rehabilitation approaches could represent an attractive therapeutic option.

## Supplementary Material

Supplemental Material

## Data Availability

Data sharing is not applicable to this article as no new data were created or analysed in this study.
