# Supplementary material for: Clinical assessment of endothelial function in convalescent COVID-19 patients: a meta-analysis with meta-regressions
Source: Ann Med. 2022 Nov 16;54(1):3234–49. doi: 10.1080/07853890.2022.2136403 (PMC9673781; doi:10.1080/07853890.2022.2136403)
Supplement: Supplemental Material [file IANN_A_2136403_SM4572.doc]

**Supplemental online material**

**Clinical assessment of endothelial function in convalescent COVID-19 patients:**

**A meta-analysis with meta-regressions.**

Pasquale Ambrosino, **MD PhD** ^1*^ Stefano Sanduzzi Zamparelli, **MD** ^2*^ Marco Mosella, **MD** ^3^

Roberto Formisano, **MD** ^1^ Antonio Molino, **MD** ^2^ Giorgio Alfredo Spedicato, **PhD FCAS FSA CSPA CStat** ^4^ Antimo Papa, **MD** ^1^ Andrea Motta, **PhD** ^5^ Matteo Nicola Dario Di Minno, **MD PhD** ^2**^

Mauro Maniscalco, **MD PhD** ^2,6**^

**The two Authors equally contributed to the manuscript.*

***The two Authors share co-seniorship.*

^1^ Istituti Clinici Scientifici Maugeri IRCCS, Cardiac Rehabilitation Unit of Telese Terme Institute, Italy;

^2^ Department of Clinical Medicine and Surgery, Federico II University, Naples, Italy;

^3^ Istituti Clinici Scientifici Maugeri IRCCS, Neurological Rehabilitation Unit of Telese Terme Institute, Italy;

^4^ Department of Data Analytics and Actuarial Science, Unipol Group, Bologna, Italy;

^5^ Institute of Biomolecular Chemistry, National Research Council, Pozzuoli, Naples, Italy;

^6^ Istituti Clinici Scientifici Maugeri IRCCS, Pulmonary Rehabilitation Unit of Telese Terme Institute, Italy.

**Table of contents:**

| **Supplemental Table 1** | **PubMed search (June 9, 2022).** |
| --- | --- |
| **Supplemental Table 2** | **Medications used by convalescent coronavirus disease 2019 (COVID-19) patients and controls in included datasets.** |
| **Supplemental Table 3** | **Enrolment of convalescent coronavirus disease 2019 (COVID-19) patients in included studies.** |
| **Supplemental Table 4** | **Ultrasound machine and edge detection software for flow-mediated dilation (FMD) evaluation in included studies.** |
| **Supplemental Table 5** | **Assessment of quality of studies (Newcastle-Ottawa scale).** |
| **Supplemental Table 6** | **Assessment of publication bias.** |
| **Supplemental Table 7** | **Sensitivity and subgroup multilevel analyses for studies evaluating flow-mediated dilation (FMD) in convalescent coronavirus disease 2019 (COVID-19) patients and controls. Panel A: “high quality” studies (Newcastle-Ottawa Scale ≥ 7); Panel B: studies specifically excluding participants with any cardiovascular risk factor or history of coronary artery disease; Panel C: exclusion of studies on pediatric populations; Panel D: studies using an automatic edge detection software for FMD assessment; Panel E: studies evaluating convalescent patients within the first 3 months of severe acute respiratory syndrome coronavirus 2 (SARS-CoV-2) infection; Panel F: studies evaluating COVID-19 participants more than 3 months after recovery.** |
| **Supplemental Table 8** | **Univariate meta-regressions. Impact of differences (Δ) in key clinical and demographic variables and impact of coronavirus disease 2019 (COVID-19) related variables on the difference in flow-mediated dilation (FMD) between cases and controls.** |
| **Supplemental Table 9** | **Multivariate meta-regressions. Impact of differences (Δ) in key clinical and demographic variables on the difference in flow-mediated dilation (FMD) between cases and controls, assuming that modifiers are held constant.** |
| **Supplemental Figure 1** | **Sensitivity analysis for unmeasured confounding.** |

**Supplemental Table 1. PubMed search (June 9, 2022).**

| **Search terms** | **Number of results** |
| --- | --- |
| (severe acute respiratory syndrome coronavirus 2 OR SARS-CoV-2 OR coronavirus disease 2019 OR COVID-19 OR long COVID OR post-COVID-19 OR COVID long-haulers) | 266,464 |
| (severe acute respiratory syndrome coronavirus 2 OR SARS-CoV-2 OR coronavirus disease 2019 OR COVID-19 OR long COVID OR post-COVID-19 OR COVID long-haulers) AND (flow-mediated dilation) | 32 |
| (severe acute respiratory syndrome coronavirus 2 OR SARS-CoV-2 OR coronavirus disease 2019 OR COVID-19 OR long COVID OR post-COVID-19 OR COVID long-haulers) AND (flow-mediated dilation OR FMD) | 46 |
| (severe acute respiratory syndrome coronavirus 2 OR SARS-CoV-2 OR coronavirus disease 2019 OR COVID-19 OR long COVID OR post-COVID-19 OR COVID long-haulers) AND (flow-mediated dilation OR FMD OR nitrate-mediated dilation) | 46 |
| (severe acute respiratory syndrome coronavirus 2 OR SARS-CoV-2 OR coronavirus disease 2019 OR COVID-19 OR long COVID OR post-COVID-19 OR COVID long-haulers) AND (flow-mediated dilation OR FMD OR nitrate-mediated dilation OR NMD) | 94 |
| (severe acute respiratory syndrome coronavirus 2 OR SARS-CoV-2 OR coronavirus disease 2019 OR COVID-19 OR long COVID OR post-COVID-19 OR COVID long-haulers) AND (flow-mediated dilation OR FMD OR nitrate-mediated dilation OR NMD OR endothelium-dependent dilation) | 94 |
| (severe acute respiratory syndrome coronavirus 2 OR SARS-CoV-2 OR coronavirus disease 2019 OR COVID-19 OR long COVID OR post-COVID-19 OR COVID long-haulers) AND (flow-mediated dilation OR FMD OR nitrate-mediated dilation OR NMD OR endothelium-dependent dilation OR endothelium-independent dilation) | 94 |

**Supplemental Table 2. Medications used by convalescent coronavirus disease 2019 (COVID-19) patients and controls in included datasets.**

| **Study** | **Subjects**  **(n)** | **β-blockers**  **n (%)** | **CCB**  **n (%)** | **RAS inhibitors**  **n (%)** | **Diuretics**  **n (%)** | **Statins**  **n (%)** | **Antiplatelets**  **n (%)** | **Anticoagulants**  **n (%)** | **Insulin**  **n (%)** | **Oral antidiabetics**  **n (%)** |
| --- | --- | --- | --- | --- | --- | --- | --- | --- | --- | --- |
| Ambrosino 2021 | 133 COVID-19 | 42 (31.6) | - | 31 (23.3) | - | 39 (29.3) | 20 (15.0) | - | 17 (12.8) | 16 (12.0) |
|  | 133 controls | 55 (41.4) | - | 23 (17.3) | - | 23 (17.3) | 32 (24.1) | - | 14 (10.5) | 32 (24.1) |
| Çiftel 2021 | 38 COVID-19 | - | - | - | - | - | - | - | 0 | 0 |
|  | 38 controls | - | - | - | - | - | - | - | 0 | 0 |
| Ergül 2021 | 63 COVID-19 | - | - | - | - | - | - | - | - | - |
|  | 29 controls | - | - | - | - | - | - | - | - | - |
| Gao 2022 (a) | 86 COVID-19 | - | - | - | - | - | - | - | - | - |
|  | 28 controls | - | - | - | - | - | - | - | 0 | 0 |
| Gao 2022 (b) | 86 COVID-19 | - | - | - | - | - | - | - | - | - |
|  | 30 controls | - | - | - | - | - | - | - | - | - |
| Jud 2021 (a) | 14 COVID-19 | 3 (21.4) | 2 (14.3) | 5 (35.7) | 1 (7.1) | 0 | 0 | 2 (14.3) | 0 | 0 |
|  | 14 controls | 0 | 0 | 0 | 0 | 0 | 0 | 0 | 0 | 0 |
| Jud 2021 (b) | 14 COVID-19 | 3 (21.4) | 2 (14.3) | 5 (35.7) | 1 (7.1) | 0 | 0 | 2 (14.3) | 0 | 0 |
|  | 14 controls | 9 (64.3) | 5 (35.7) | 9 (64.3) | 4 (28.6) | 10 (71.4) | 9 (64.3) | 3 (21.4) | 0 | 6 (42.9) |
| Lambadiari 2021 (a) | 70 COVID-19 | - | - | - | - | - | - | - | 0 | 0 |
|  | 70 controls | - | - | - | - | - | - | - | 0 | 0 |
| Lambadiari 2021 (b) | 70 COVID-19 | - | - | - | - | - | - | - | 0 | 0 |
|  | 70 controls | - | - | - | - | - | - | - | 0 | 0 |
| Mansiroglu 2022 | 80 COVID-19 | - | - | - | - | - | - | - | - | - |
|  | 81 controls | - | - | - | - | - | - | - | - | - |
| Nandadeva 2021 (a) | 8 COVID-19 | 0 | 0 | 0 | 0 | 0 | 0 | 0 | 0 | 0 |
|  | 12 controls | 0 | 0 | 0 | 0 | 0 | 0 | 0 | 0 | 0 |
| Nandadeva 2021 (b) | 8 COVID-19 | 0 | 0 | 0 | 0 | 0 | 0 | 0 | 0 | 0 |
|  | 12 controls | 0 | 0 | 0 | 0 | 0 | 0 | 0 | 0 | 0 |
| Oikonomu 2022 (a) | 55 COVID-19 | - | - | - | - | - | - | - | - | - |
|  | 55 controls | - | - | - | - | - | - | - | - | - |
| Oikonomu 2022 (a) | 55 COVID-19 | - | - | - | - | - | - | - | - | - |
|  | 55 controls | - | - | - | - | - | - | - | - | - |
| Ratchford 2021 | 11 COVID-19 | 0 | - | 0 | 0 | 0 | - | - | 0 | 0 |
|  | 20 controls | 0 | - | 0 | 0 | 0 | - | - | 0 | 0 |
| Riou 2021 | 27 COVID-19 | - | - | - | - | - | - | - | - | - |
|  | 9 controls | - | - | - | - | - | - | - | - | - |
| Skow 2022 | 23 COVID-19 | 0 | 0 | 0 | 0 | 0 | 0 | 0 | 0 | 0 |
|  | 13 controls | 0 | 0 | 0 | 0 | 0 | 0 | 0 | 0 | 0 |

**CCB: calcium channel blockers; RAS: renin angiotensin system. The minus sign indicates that the information has not been specifically provided and/or cannot be extrapolated from the full-text article. For studies specifically excluding diabetic patients, the non-use of oral antidiabetics and insulin has been assumed.**

**Supplemental Table 3. Enrolment of convalescent coronavirus disease 2019 (COVID-19) patients in included studies.**

| **Study** | **Period** | **Follow-up after infection** | **Country** |
| --- | --- | --- | --- |
| Ambrosino 2021 | Between December 2020 to June 2021 | Within 2 months of swab test negativization | Italy |
| Çiftel 2021 | Between September 2020 to March 2021 | 6-8 weeks after SARS-CoV-2 infection | Turkey |
| Ergül 2021 | Between November 2020 and January 2021 | 2 months after hospital discharge | Turkey |
| Gao 2022 | Between December 2020 and January 2021 | A median of 327 days after recovery | China |
| Jud 2021 | Between March 2020 and April 2020 | - | Austria |
| Lambadiari 2021 | Between September 2020 and January 2021 | 4 months after SARS-CoV-2 infection | Greece |
| Mansiroglu 2022 | Between October 2020 and February 2021 | Within 6 months of diagnosis | Turkey |
| Nandadeva 2021 | - | A minimum of 4 weeks from diagnosis | USA |
| Oikonomou 2022 | Between November 2020 and March 2021 | 28 days and 6 months after hospital discharge | Greece |
| Ratchford 2021 | - | 3-4 weeks after SARS-CoV-2 infection | USA |
| Riou 2021 | Between March 2020 and April 2020 | 3 months after SARS-CoV-2 infection | France |
| Skow 2022 | Between January 2022 and February 2022 | Within 6 weeks of COVID-19 diagnosis | USA |

**Supplemental Table 4. Ultrasound machine and edge detection software for flow-mediated dilation (FMD) evaluation in included studies.**

| **Study** | **Ultrasound machine** | **Software** |
| --- | --- | --- |
| Ambrosino 2021 | Vivid E95 (GE Healthcare, Illinois, USA) 10 MHz linear probe | Cardiovascular Suite, QUIPU Srl, Pisa, Italy |
| Çiftel 2021 | Vivid S60N (GE Healthcare, Illinois, USA) 12 MHz linear probe | No |
| Ergül 2021 | Unspecified ultrasound machine  5-13 MHz linear probe | No |
| Gao 2022 | Vivid E95 (GE Healthcare, Illinois, USA) 4-8 MHz linear probe | No |
| Jud 2021 | ACUSON S2000TM (Siemens Healthcare Corp., Erlangen, Germany)  8-13 MHz linear probe | No |
| Lambadiari 2021 | Vivid E95 (GE Healthcare, Illinois, USA) 10 MHz linear probe | No |
| Mansiroglu 2022 | M4S-RS (GE Healthcare, Illinois, USA) 7.5 MHz linear probe | No |
| Nandadeva 2021 | Logiq P5 (GE Healthcare, Illinois, USA) 11 MHz linear probe | LabView, National Instruments, Austin, TX |
| Oikonomou 2022 | Vivid E (GE Healthcare, Illinois, USA) 5-13 MHz linear probe | No |
| Ratchford 2021 | Logiq P5 (GE Healthcare, Illinois, USA) 4-12 MHz linear probe | Cardiovascular Suite, QUIPU Srl, Pisa, Italy |
| Riou 2021 | - | No |
| Skow 2022 | Logiq P5 (GE Healthcare, Illinois, USA) 11 MHz linear probe | LabView, National Instruments, Austin, TX |

**Supplemental Table 5. Assessment of quality of studies (Newcastle-Ottawa scale).**

| **Study** | **Adequate case definition** | **Representativeness of the cases** | **Selection of controls** | **Definition of controls** | **Comparability of cases and**  **controls on the basis of the**  **design or analysis*** | **Ascertainment of exposure** | **Same method of ascertainment**  **for cases and controls** | **Non-response rate** | **Quality** |
| --- | --- | --- | --- | --- | --- | --- | --- | --- | --- |
| Ambrosino 2021 | **🟋** | **🟋** | **-** | **🟋** | **🟋🟋** | **🟋** | **🟋** | **🟋** | 8 |
| Çiftel 2021 | **🟋** | **-** | **🟋** | **🟋** | **🟋🟋** | **🟋** | **🟋** | **🟋** | 8 |
| Ergül 2021 | **🟋** | **🟋** | **🟋** | **🟋** | **-** | **🟋** | **🟋** | **🟋** | 7 |
| Gao 2022 | **🟋** | **-** | **-** | **🟋** | **🟋** | **🟋** | **🟋** | **🟋** | 6 |
| Jud 2021 | **🟋** | **-** | **-** | **🟋** | **-** | **🟋** | **🟋** | **🟋** | 5 |
| Lambadiari 2021 | **🟋** | **🟋** | **🟋** | **🟋** | **🟋** | **🟋** | **🟋** | **🟋** | 8 |
| Mansiroglu 2022 | **🟋** | **-** | **-** | **🟋** | **🟋🟋** | **🟋** | **🟋** | **🟋** | 7 |
| Nandadeva 2021 | **🟋** | **-** | **-** | **🟋** | **-** | **🟋** | **🟋** | **🟋** | 5 |
| Oikonomou 2022 | **🟋** | **-** | **-** | **🟋** | **🟋🟋** | **🟋** | **🟋** | **🟋** | 7 |
| Ratchford 2021 | **🟋** | **-** | **-** | **🟋** | **-** | **🟋** | **🟋** | **🟋** | 5 |
| Riou 2021 | **🟋** | **-** | **-** | **🟋** | **🟋** | **🟋** | **🟋** | **🟋** | 6 |
| Skow 2022 | **🟋** | **-** | **🟋** | **🟋** | **🟋🟋** | **🟋** | **🟋** | **🟋** | 8 |

***A maximum of 2 stars can be allotted in this category: 🟋if enrolling controls matched to cases for age and sex; 🟋🟋 if enrolling controls matched to cases for any additional factor. N/A: not applicable.**

**Supplemental Table 6. Assessment of publication bias.**

| **Egger’s regression intercept** | Intercept | -0.99600 |  |
| --- | --- | --- | --- |
|  | Standard error | 1.98448 |  |
|  | 95% lower limit | -5.22581 |  |
|  | 95% upper limit | 3.23381 |  |
|  | t-value | 0.50190 |  |
|  | P value | 0.62303 |  |
| **Begg and Mazumdar** | **Kendall’s S statistic (P-Q)** | -14.00000 |  |
|  | **Kendall’s tau without continuity correction** |  |  |
|  | tau | -0.10370 |  |
|  | Z-value for tau | 0.57670 |  |
|  | P-value | 0.56414 |  |
|  | **Kendall’s tau with continuity correction** |  |  |
|  | tau | -0.09630 |  |
|  | Z-value for tau | 0.53550 |  |
|  | P-value | 0.59230 |  |

| **Duvall and Tweedie’s trim and fill** | **Studies**  **trimmed** | **Point**  **estimate** | **Lower**  **limit** | **Upper**  **limit** | **Q value** |
| --- | --- | --- | --- | --- | --- |
| Observed values | - | -2.313 | -3.186 | -1.441 | 183.84382 |
| Adjusted values (on the left) | 1 | -2.501 | -3.373 | -1.630 | 196.77111 |
| Adjusted values (on the right) | 0 | -2.313 | -3.186 | -1.441 | 183.84382 |

**Supplemental Table 7. Sensitivity and subgroup multilevel analyses for studies evaluating flow-mediated dilation (FMD) in convalescent coronavirus disease 2019 (COVID-19) patients and controls. Panel A: “high quality” studies (Newcastle-Ottawa Scale ≥ 7); Panel B: studies specifically excluding participants with any cardiovascular risk factor or history of coronary artery disease; Panel C: exclusion of studies on pediatric populations; Panel D: studies using an automatic edge detection software for FMD assessment; Panel E: studies evaluating convalescent patients within the first 3 months of severe acute respiratory syndrome coronavirus 2 (SARS-CoV-2) infection; Panel F: studies evaluating COVID-19 participants more than 3 months after recovery.**

|  | *N. of studies* | *N. of datasets* | *N. of patients* | *Effect size* |
| --- | --- | --- | --- | --- |
| **SENSITIVITY**  **ANALYSES** | **A. “High quality” studies** | | | |
|  | 7 | 9 | 517 COVID-19  489 controls | MD: -1.93 (95%CI: -2.90, -0.96); **P<0.0001**  I^2^=96.0%; P<0.0001  PI: -4.68, 0.82 |
|  | **B. Exclusion of participants with cardiovascular risk factors or history of events** | | | |
|  | 5 | 6 | 202 COVID-19  188 controls | MD: -1.74 (95%CI: -2.96, -0.51); **P=0.010**  I^2^=98.6%; P<0.0001  PI: -4.86, 1.38 |
|  | **C. Exclusion of pediatric populations** | | |  |
|  | 11 | 16 | 606 COVID-19  624 controls | MD: -2.25 (95%CI: -3.41, -1.09); **P<0.0001**  I^2^=98.7%; P<0.0001  PI: -6.23, 1.73 |
|  | **D. Use of an automatic edge detection software** | | | |
|  | 4 | 5 | 227 COVID-19  213 controls | MD: -1.54 (95%CI: -2.93, -0.14); **P=0.031**  I^2^=99.0%; P<0.0001  PI: -4.87, 1.80 |
| **SUBGROUP**  **ANALYSES** | **E. Follow-up ≤ 3 months** | | | |
|  | 9 | 11 | 408 COVID-19  383 controls | MD: -2.21 (95%CI: -3.71, -0.72); **P=0.004**  I^2^=98.5%; P<0.0001  PI: -6.55, 2.13 |
|  | **F. Follow-up > 3 months** | | | |
|  | 3 | 5 | 211 COVID-19  253 controls | MD: -2.44 (95%CI: -4.30, -0.59); **P=0.009**  I^2^=98.5%; P<0.0001  PI: -6.61, 1.72 |

**N: number; MD: mean difference; 95%CI: 95% confidence interval; PI: prediction interval.**

**Supplemental Table 8. Univariate meta-regressions. Impact of differences (Δ) in key clinical and demographic variables and impact of coronavirus disease 2019 (COVID-19) related variables on the difference in flow-mediated dilation (FMD) between cases and controls.**

| **Independent variable** | **Main results** | **Proportion of total between-study**  **variance explained by the model** | **Goodness of fit** |
| --- | --- | --- | --- |
| Δ _% of males_ | Z-score: 0.41, P=0.680 | R^2^ analog: 0.00 | Q: 145.48, df: 14; P<0.001 |
| Δ _Age_ | Z-score: 0.74, P=0.462 | R^2^ analog: 0.00 | Q: 167.16, df: 14; P<0.001 |
| Δ _BMI_ | Z-score: -0.05, P=0.962 | R^2^ analog: 0.00 | Q: 86.95, df: 8; P<0.001 |
| Δ _% of diabetes_ | **Z-score: -2.51, P=0.012** | R^2^ analog: 0.19 | Q: 116.91, df: 13; P<0.001 |
| Δ _% of hypertension_ | **Z-score: -2.14, P=0.033** | R^2^ analog: 0.24 | Q: 106.45, df: 13; P<0.001 |
| Δ _% of hypercholesterolemia_ | Z-score: -0.75, P=0.454 | R^2^ analog: 0.00 | Q: 147.66, df: 13; P<0.001 |
| Δ _% of smoking_ | Z-score: -1.36, P=0.173 | R^2^ analog: 0.00 | Q: 103.39, df: 8; P<0.001 |
| Δ _% of CAD_ | **Z-score: -2.21, P=0.027** | R^2^ analog: 0.14 | Q: 126.77, df: 13; P<0.001 |
| Critical disease | Z-score: -1.20, P=0.229 | R^2^ analog: 0.00 | Q: 90.47, df: 8; P<0.001 |
| Hospitalization | Z-score: -1.43, P=0.151 | R^2^ analog: 0.11 | Q: 99.40, df: 9; P<0.001 |
| LHS | N/A | N/A | N/A |
| High-flow O_2_ | N/A | N/A | N/A |
| Mechanical ventilation | Z-score: -0.95, P=0.341 | R^2^ analog: 0.00 | Q: 63.80, df: 5; P<0.001 |
| ICU | Z-score: -1.32, P=0.187 | R^2^ analog: 0.00 | Q: 49.50, df: 4; P<0.001 |
| WBC | N/A | N/A | N/A |
| CRP | Z-score: 0.20, P=0.839 | R^2^ analog: 0.00 | Q: 41.52, df: 5; P<0.001 |
| PASC | **Z-score: -2.09, P=0.037** | R^2^ analog: 0.45 | Q: 49.04, df: 8; P<0.001 |
| Δ _% of β-blockers_ | Z-score: -0.53, P=0.596 | R^2^ analog: 0.00 | Q: 74.78, df: 5; P<0.001 |
| Δ _% of CCB_ | Z-score: -0.55, P=0.581 | R^2^ analog: 0.00 | Q: 58.69, df: 3; P<0.001 |
| Δ _% of RAS inhibitors_ | Z-score: -0.60, P=0.550 | R^2^ analog: 0.00 | Q: 71.15, df: 5; P<0.001 |
| Δ _% of diuretics_ | Z-score: -0.84, P=0.401 | R^2^ analog: 0.00 | Q: 57.14, df: 4; P<0.001 |
| Δ _% of statins_ | Z-score: -1.52, P=0.128 | R^2^ analog: 0.15 | Q: 60.24, df: 5; P<0.001 |
| Δ _% of antiplatelets_ | Z-score: -0.90, P=0.370 | R^2^ analog: 0.00 | Q: 71.32, df: 4; P<0.001 |
| Δ _% of anticoagulants_ | Z-score: -0.23, P=0.819 | R^2^ analog: 0.00 | Q: 60.98, df: 3; P<0.001 |
| Δ _% of insulin_ | Z-score: -1.20, P=0.232 | R^2^ analog: 0.00 | Q: 94.80, df: 8; P<0.001 |
| Δ _% of oral antidiabetics_ | Z-score: -0.94, P=0.349 | R^2^ analog: 0.00 | Q: 108.12, df: 8; P<0.001 |

**BMI: body mass index; CAD: coronary artery disease; LHS: length of hospital stays; O_2_: oxygen; ICU: intensive care unit; WBC: white blood cells; CRP: C-reactive protein; PASC: post-acute sequelae of COVID-19; CCB: calcium channel blockers; RAS: renin angiotensin system; N/A: not assessed because of the limited number of studies reporting this co-variate.**

**Supplemental Table 9. Multivariate meta-regressions. Impact of differences (Δ) in key clinical and demographic variables on the difference in flow-mediated dilation (FMD) between cases and controls, assuming that modifiers are held constant.**

| **Independent variable** | **Modifiers** | **Main results** | **Proportion of total between-study variance explained by the model** | **Goodness of fit** |
| --- | --- | --- | --- | --- |
| Δ _% of diabetes_ | Δ _Males (%)_  Δ _Age_  Δ _BMI_ | **Z-score: -3.62; P<0.001** | R^2^ analog: 0.68 | Q: 19.05, df: 5; P=0.0019 |
| Δ _% of hypertension_ | Δ _Males (%)_  Δ _Age_  Δ _BMI_ | **Z-score: -2.56; P=0.011** | R^2^ analog: 0.35 | Q: 34.03, df: 5; P<0.001 |
| Δ _% of CAD_ | Δ _Males (%)_  Δ _Age_  Δ _BMI_ | **Z-score: -2.16; P=0.031** | R^2^ analog: 0.19 | Q: 43.46, df: 5; P<0.001 |

**BMI: body mass index; CAD: coronary artery disease; df: degree of freedom.**

**Supplemental Figure 1. Sensitivity analysis for unmeasured confounding.**


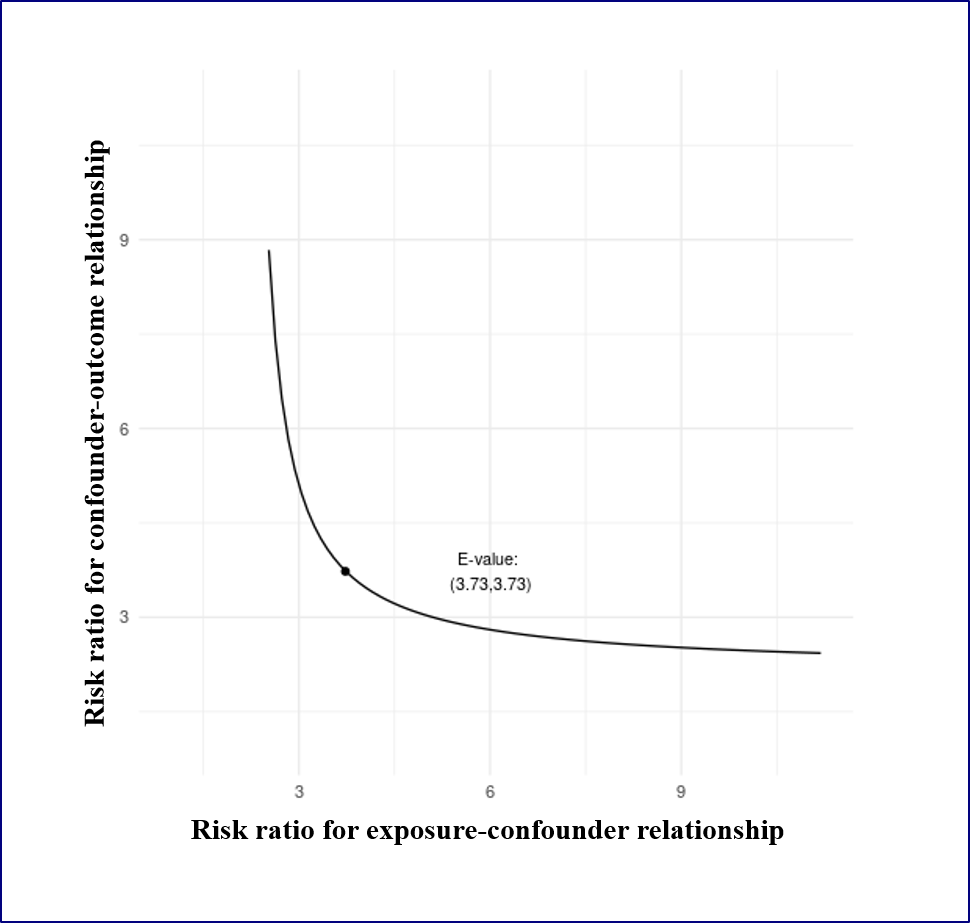


**Each point along the curve defines a joint relationship between the two sensitivity parameters that could potentially explain away the estimated effect. If one of the two parameters is smaller than the E-value, the other must be larger, as defined by the plotted curve.**
